# Time course of cardiometabolic alterations in a high fat high sucrose diet mice model and improvement after GLP-1 analog treatment using multimodal cardiovascular magnetic resonance

**DOI:** 10.1186/s12968-015-0198-x

**Published:** 2015-11-06

**Authors:** Inès Abdesselam, Pauline Pepino, Thomas Troalen, Michael Macia, Patricia Ancel, Brice Masi, Natacha Fourny, Bénédicte Gaborit, Benoît Giannesini, Frank Kober, Anne Dutour, Monique Bernard

**Affiliations:** Aix-Marseille Université, CNRS, CRMBM, UMR7339, 27, Bd Jean Moulin, 13385 Marseille, France; Aix-Marseille Université, NORT, Inserm U1062/Inra1260, 13385 Marseille, France; Endocrinology, Metabolic diseases and nutrition, CHU Nord, Marseille, France

**Keywords:** Cardiovascular magnetic resonance, Proton-magnetic resonance spectroscopy, Obesity, Diabetes, DIO mice model, Longitudinal study

## Abstract

**Background:**

Cardiovascular complications of obesity and diabetes are major health problems. Assessing their development, their link with ectopic fat deposition and their flexibility with therapeutic intervention is essential. The aim of this study was to longitudinally investigate cardiac alterations and ectopic fat accumulation associated with diet-induced obesity using multimodal cardiovascular magnetic resonance (CMR) in mice. The second objective was to monitor cardiac response to exendin-4 (GLP-1 receptor agonist).

**Methods:**

Male C57BL6R mice subjected to a high fat (35 %) high sucrose (34 %) (HFHSD) or a standard diet (SD) during 4 months were explored every month with multimodal CMR to determine hepatic and myocardial triglyceride content (HTGC, MTGC) using proton MR spectroscopy, cardiac function with cine cardiac MR (CMR) and myocardial perfusion with arterial spin labeling CMR. Furthermore, mice treated with exendin-4 (30 μg/kg SC BID) after 4 months of diet were explored before and 14 days post-treatment with multimodal CMR.

**Results:**

HFHSD mice became significantly heavier (+33 %) and displayed glucose homeostasis impairment (1-month) as compared to SD mice, and developed early increase in HTGC (1 month, +59 %) and MTGC (2-month, +63 %). After 3 months, HFHSD mice developed cardiac dysfunction with significantly higher diastolic septum wall thickness (sWtnD) (1.28 ± 0.03 mm vs. 1.12 ± 0.03 mm) and lower cardiac index (0.45 ± 0.06 mL/min/g vs. 0.68 ± 0.07 mL/min/g, *p* = 0.02) compared to SD mice. A significantly lower cardiac perfusion was also observed (4 months:7.5 ± 0.8 mL/g/min vs. 10.0 ± 0.7 mL/g/min, *p* = 0.03). Cardiac function at 4 months was negatively correlated to both HTGC and MTGC (*p* < 0.05). 14-day treatment with Exendin-4 (Ex-4) dramatically reversed all these alterations in comparison with placebo-treated HFHSD. Ex-4 diminished myocardial triglyceride content (−57.8 ± 4.1 %), improved cardiac index (+38.9 ± 10.9 %) and restored myocardial perfusion (+52.8 ± 16.4 %) under isoflurane anesthesia. Interestingly, increased wall thickness and hepatic steatosis reductions were independent of weight loss and glycemia decrease in multivariate analysis (*p* < 0.05).

**Conclusion:**

CMR longitudinal follow-up of cardiac consequences of obesity and diabetes showed early accumulation of ectopic fat in mice before the occurrence of microvascular and contractile dysfunction. This study also supports a cardioprotective effect of glucagon-like peptide-1 receptor agonist.

**Electronic supplementary material:**

The online version of this article (doi:10.1186/s12968-015-0198-x) contains supplementary material, which is available to authorized users.

## Background

With the worldwide alarming increase in obesity, cardiovascular complications of obesity and diabetes are a major public health problem. Indeed, obesity and diabetes are closely inter-related risk factors for heart disease [1, 2]. First, there is a well-characterized association between obesity and type 2 diabetes. Obesity leads to a 20-fold increase in the prevalence of type 2 diabetes in women and 10-fold in men [1]. Second, chronic hyperglycemia may induce diabetic cardiomyopathy [3–6]. It can lead to cardiac damage and microvascular complications independently of coronary artery disease and hypertension. Third, an inverse correlation between myocardial blood flow reserve and poor glycemic control in type 2 diabetes has been clearly demonstrated [7].

Ectopic fat deposition often results from dysfunction of subcutaneous adipose tissue and imbalance between fatty acid uptake and oxidation rate. It is considered to contribute to organ dysfunction via an effect commonly described as lipotoxicity [8]. Many studies have reported that lipid accumulation in the heart and in the liver increases in obese and diabetic mice, and that this increase is associated with diastolic function impairment [9–11]. In addition, many studies in animal models have demonstrated that cardiac ectopic fat accumulation is causally linked with cardiac dysfunction [12, 13]. Glucotoxicity and lipotoxicity are both well recognized initiators of heart diseases [13, 14], but their chronological effect *in vivo* under different metabolic conditions still needs to be clarified.

For this reason, there is increasing need for new methods to better characterize cardiac alterations and ectopic fat development and to analyze the impact of new potential treatments against these complex and interrelated metabolic conditions.

Cardiovascular magnetic resonance (CMR) techniques are potentially valuable tools, which have shown excellent ability to provide useful data on fat distribution and cardiac function in humans [15, 16]. In mice, proton MR spectroscopy (^1^H-MRS), in particular, gives access to the molecular content of cardiac or hepatic triglycerides (TG), and it has been shown to provide good accuracy when compared to gold standard biochemical assays [11, 17]. Considering adverse effects of diabetes on microvessels, there is strong evidence suggesting that the integrity of the vascular endothelium is altered in this disease eventually resulting in myocardial injury [18, 19]. Moreover, Naresh et al., showed reduced myocardial perfusion reserve using dual-contrast first-pass CMR sequence in mice after 24 weeks of high-fat diet. However, whether this reduction is associated to high-fat diet-induced obesity or diabetes development hasn’t been explored so far. Accordingly, microvascular abnormality deserves specific mention, and better knowledge on functional alterations of the coronary microcirculation may in the future serve in evaluating and monitoring potential therapeutic regulation approaches of endothelial dysfunction in T2D. CMR is playing an expanding role in the non-invasive assessment of myocardial blood flow. Arterial spin labeling (ASL) CMR techniques in particular appear as a powerful and direct tool for the assessment of murine myocardial perfusion without any injection of contrast agents [20–23]. They can further be performed repeatedly and are therefore a good candidate for longitudinal tracking.

To date there are few longitudinal multi-modal in vivo studies combining the assessment of all these parameters to study the disease progression. Here, we implemented a protocol providing combined assessment of several parameters including global myocardial function, ectopic accumulation of triglycerides in non-adipose tissues (heart and liver steatosis) and myocardial perfusion and applied it to a HFHSD-induced mouse model of obesity and diabetes. The combination of the entire set of advanced CMR and hepatic techniques into a single protocol was possible mainly by using the rapid cine-ASL perfusion CMR approach and a two-slice cine-CMR sequence that was found to provide good global function approximations earlier [24]. The 30–45 min protocol was repeated in a 4-month longitudinal follow-up during development of glucose intolerance in the HFHSD mice. The HFHSD model was chosen because it is considered to mimic best the human western diet with comparable consequences on body composition changes, impaired glucose tolerance and insulin sensitivity, hepatic steatosis, cardiac structure and function [25].

In an additional session, we assessed the effects of Exendin 4 (GLP-1 receptor agonist), administered during a short time (15 days, with small weight effect) to the animals in order to assess its effect on cardiac perfusion and function, on ectopic fat development as well as on total body fat. Incretins, glucagon-like peptide-1 (GLP-1) receptor agonists are new pleiotropic drugs widely used in type 2 diabetic patients. These drugs actually improve glycemic profile, decrease glucagon secretion and increase satiety via their action on central nervous system. Besides, emerging evidence suggests beneficial effects of these molecules on cardiac structure and function. GLP-1 and GLP-1 receptor agonists would indeed decrease both inflammatory state [26] and blood pressure [27], improve endothelial [28, 29] and cardiac function in ischemia reperfusion infarction model [30]. However, short time effect of Exendin 4 on cardiac function and on ectopic fat development has been rarely studied [31, 32].

## Methods

### Animals

All animal procedures were approved by the Animal experiment ethic committee of Aix-Marseille University (n°40-10102012) and were in conformity with the European Convention for the protection of animals used for experimental purposes.

Fifty-six C57BL/6 J Rj eight-week-old male mice were purchased from Janvier labs (France). Animals were housed 2 weeks before experimentation in a controlled environment under standard laboratory conditions: a 12 h-12 h light–dark cycle and room temperature maintained at 24 °C. The mice had *ad libitum* access to water and food.

### Diet and exendin-4 treatment experimental protocol

**Fig. 1:**
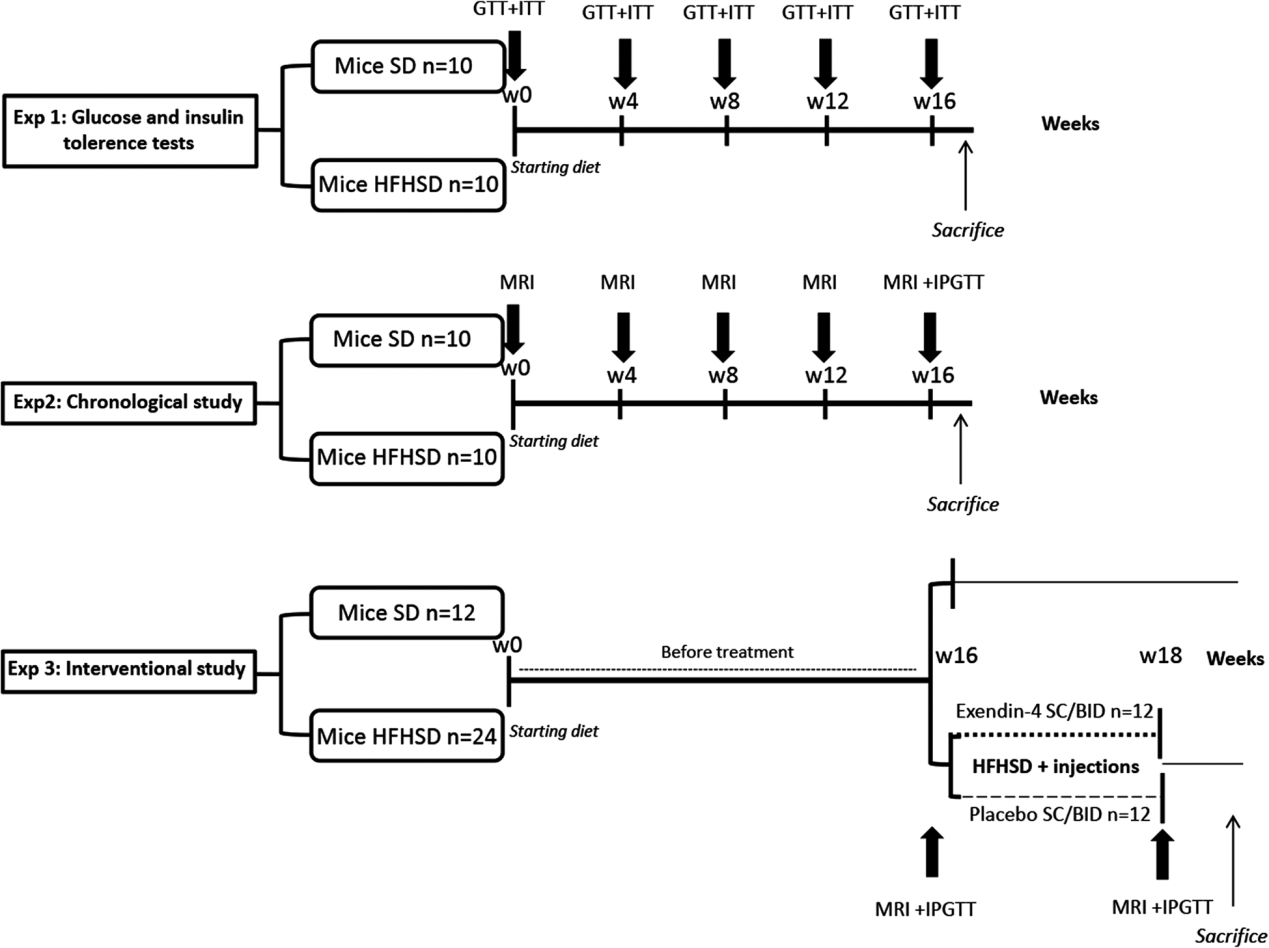
Flowchart of mice in different diet groups and treatment. HFHSD mice (C57BL6J on High fat high sucrose diet) and SD mice (C57BL6J on control diet) were kept on diet for 16 weeks in the first experiment (glucose homeostasis study), both glucose and insulin tolerance tests were performed every month in order to determine time of impaired glucose homeostasis. In the second experiment (chronological study), a magnetic resonance (MR) examination was performed every month and intraperitoneal glucose tolerance test was done at the end of experiment (16 weeks) before sacrifice. In the third experiment (experimental study), SD mice were kept on standard diet only for 16 weeks until MR examination and IPGTT. Mice fed HFHSD underwent MR examination and IPGTT at 16 weeks, before treatment with exendin-4 or physiological serum during 14 days and then underwent once again MR examination and IPGTT after treatment

The study was divided into three experimental protocols (Fig. 1). In the first protocol, we performed a longitudinal follow-up of glucose homeostasis alteration in ten mice fed a high-fat high-sucrose diet (HFHSD, 35 % fat, 34 % carbohydrate, 22 % protein) for 16 weeks compared to ten mice fed a standard chow diet (SD, 60 % carbohydrate, 3 % fat, 16 % protein). Composition of the diet is detailed in Additional file 1. In the second protocol, we performed another monthly longitudinal follow-up of cardiac alterations and ectopic fat deposition including: CMR, liver MRS and whole body fat mass MRI. For this purpose, ten mice were fed a SD, and 10 mice were fed a HFHSD for 16 weeks. In the third protocol, 12 mice were fed a SD, and 24 mice were fed HFHSD during 16 weeks and were then treated with Exendin-4 (30 μg/Kg SC BID) or placebo (phosphate-buffered saline SC BID) for 14 days. The MR protocol as described later was performed at 4-month post diet and after 14 days of exendin-4 treatment. Glucose tolerance test and insulin measurement were performed at the end of the experiment. Weight and glucose level were measured every month in each experimental protocol.

### CMR measurements

All CMR scanning was performed on a Bruker Biospec Avance small animal MRI system equipped with a 4.7 Tesla magnet (Bruker, Ettlingen Germany). CMR and ^1^H MRS were both performed using a proton volume resonator (diameter 60 mm, homogeneous length 80 mm) and an actively decoupled 15 mm surface receive coil (Rapid Biomedical, Wurzburg, Germany). The animals were positioned prone on the surface coil. For the CMR protocol, the animal was placed with the heart at the center of the surface coil. For the subsequent liver MRS analysis the animal was repositioned with the abdomen at the coil center.

Before the experiments, mice were sedated in an induction chamber by inhalation of a mix of 3 % isoflurane and 2 L/min pure oxygen-flow. During CMR, inhalation anesthesia was maintained at 1–2 % of isoflurane in pure oxygen at 0.6 L/min using a dedicated vaporizer (Ohmeda/General Electric, Milwaukee, WI, USA) so as to obtain regular breathing frequencies in the range of 90–100 breaths per minute. Respiration was monitored using a pressure sensor connected to an air-filled balloon positioned under the abdomen. Body temperature was monitored using a rectal probe and maintained at 37 °C using a heating blanket with hot water circulation. The electrocardiogram (ECG) signal was monitored by placing two subcutaneous electrodes in the upper limbs of the mice. The electrodes were connected to an ECG trigger unit (Rapid Biomedical, Rimpar, Germany) to record the signal and to trigger the CMR sequence. The respiratory signal was used in addition to the ECG trigger for gating the MRS scans.

### In vivo cardiac function (cine-CMR)

The assessment of myocardial mass and function by cine-CMR was performed according to the hemisphere cylinder model using one short axis area measurement and one long axis length for volume approximation. This model has recently been validated as one of the best methods to assess cardiac function in a reduced scan- and post-processing time [24]. A FLASH cine-CMR sequence (37 phases, slice thickness 1 mm, in-plane resolution 234 x 234 μm^2^, TR = 5.1 ms, TE = 1.4 ms, two averages, duration 3 min per slice) was used to acquire three perpendicular views (2-chamber, 4-chamber and mid-LV short axis).

Image segmentation and data analysis were performed using an in-house developed program running under IDL environment (ITT, Boulder, CO, USA). Epicardium, endocardium, and left ventricular lengths were manually delimited in both systole and diastole to obtain needed data for volume approximation as described previously [33].

### In vivo myocardial perfusion (Arterial spin labeling CMR)

Myocardial blood flow was quantified using a modified version of the cine-ASL technique, as described previously [23]. Cine-ASL parameters were: flip angle α = 8°, TE/TR =1.64/8 ms, field of view = 25 mm x 25 mm, matrix size = 128 x 64, resolution = 0.195 mm x 0.391 mm, excitation pulse duration = 0.5 ms, inversion pulse duration = 6 ms, imaging slice thickness = 1.5 mm, labeling slice thickness = 2.5 mm, N_*echoes*_ = 10, N_*cine*_ = 30. Images analyses were performed using a home-made program running in an IDL environment (ITT, Boulder, CO, USA) which generates perfusion maps. Using these maps, we quantified myocardial perfusion in mL/g/min.

### In vivo triglyceride accumulation (MR spectroscopy)

Cardiac ^1^H MR spectra were acquired using an ECG- and respiratory-gated Point Resolved Spectroscopy (PRESS) sequence at the systolic phase to determine the molecular content of water appearing at 4.7 ppm and of triglycerides at 1.3 ppm. The parameters were as follows: voxel size 1 x 1 x 2 mm^3^, echo time 11 ms, repetition time ranging between 700 and 1000 ms depending on the breath rate, number of averages (NA) 512. A second scan was acquired to obtain an unsaturated water peak as reference (TR = 5 s, NA = 64). Cine-CMR images in short-axis and 4-chamber views were used for voxel positioning in the basal region of the septum (Fig. 3b) far enough from the pericardial fat to avoid fat contamination.

The same sequence with slightly modified parameters (TE = 11 ms, NA = 128, and TE = 11 ms TR = 5 s, NA = 64, for the reference scan, respiratory gating) and a larger voxel size (2x2x2 mm^3^) was used for liver MRS. The voxel was placed in the anterior part of the liver (Fig. 3a).

The TR for MRS measurements of tissue TG content ranged between 700 and 1000 ms with most experiment at 1000 ms for heart and liver. To verify whether partial saturation has occurred with possible different TRs, we fitted triglyceride peak in both saturated spectra, and unsaturated reference spectra for heart an liver. Ratio of TG concentration in saturated spectra over TG concentration in unsaturated spectra is about 0.96 ± 0.15 in average in heart and liver. Therefore, underestimation of TG is inferior to 5 % and variation from one experiment to the other is weak.

Data fitting and analysis were performed using AMARES time-domain fitting routines from the MRUI package (http://www.jmrui.eu/, Fig. 3c) with a home-made software interface [34]. The TG/Water ratio was calculated to obtain the triglyceride concentration. Due to the short echo time, potential impact of T2 on the signal amplitudes was neglected.

### In vivo whole body fat mass (Magnetic resonance imaging)

For a quantitative map of adipose tissue distribution, whole-body scanning was performed using the volume resonator for radiofrequency transmit and receive. Sixty-four transverse slices were obtained across the animal body length excluding the tail with a slice thickness of 1.25 mm. High-resolution three-dimentional (turbo-spin echo) sequence was used with the following parameters: 5.530 ms echo time; 77.85 ms effective echo time; 300 ms repetition time; 2 averages; 40x40x80 mm field of view; and 128x128x64 matrix size [35]. Subcutaneous (SCAT) and visceral adipose tissue (VAT) were assessed using an automatic segmentation method based on a pixel intensity analysis of MR images [36].

### Glucose homeostasis and triglyceride measurements using biochemical analysis

Mice were sacrificed at the end of the three experimental protocols. Blood and tissue were collected when mice were sacrificed after an overnight fasting for possible ex-vivo experiments. Plasma samples were used to analyze insulin levels using an ELISA kit (Alpco, Salem, USA) and plasma triglycerides using Triglyceride Assay Kit (Chemical Company, CAYMAN). Furthermore, experimental protocol number 1 was specifically performed to analyze glucose homeostasis with IntraPeritoneal Glucose Tolerance Test (IPGTT) and Intraperitoneal Insulin Tolerance Test (IPITT) exploration alone, independently of possible repeated MR exploration-induced stress or anesthesia as it may affect in short-term metabolic parameters such as glucose and insulin [37].

IPGTT was also performed, in the second protocol at the end of experiment, after MR explorations. In the third protocol, IPGTT was performed after MR exploration as well, 16-week post-diet, and after Exendin-4 treatment.

In IPGTT and IPITT, a bolus of glucose (1 mg/g) or insulin (0.75 mU/g) was injected into the peritoneal cavity, after an over-night fasting period. Blood glucose level was measured using commercial glucometer (AccuCheck Active Glucometer, Roche, Basel, Switzerland) before glucose or insulin injection (0 min) and 15 min, 30 min, 60 min, and 120 min after injection.

After sacrifice, TGs were assayed *in vitro* in the plasma, liver and heart. In the tissues, lipids were extracted from 110 mg of liver tissue or 110 mg of heart tissue using chloroform/methanol as outlined by Folch et al. [38]. Triglycerides were measured using standard colorimetric assay (Triglyceride Assay Kit, Chemical Company, CAYMAN). Triglycerides were expressed as mg/dL.

### Statistical analysis

All data are presented as means ± SEM. Statistical analysis was performed with GraphPad Prism 5.01. Two-way ANOVA test with repeated measures was performed for all parameters such as weight, CMR cardiac parameters, ectopic lipid deposition, cardiac perfusion, and intraperitoneal glucose tolerance test, to test disease progression including effect on time and diet. Therefore, multiple comparisons test using Sidak-Bonferroni method has been achieved to show differences between groups at each time point.

Linear regression was used to evaluate the relation between ectopic fat deposition and cardiac parameters. The effect of exendin-4 treatment was analyzed with paired *t*-test or non-parametric Wilcoxon test when appropriate. Multivariate analysis was achieved using Statview 5.0 to analyze the effect of independent effect of treatment on cardiac improvement and steatosis reduction. Values of *p* <0.05 were considered statistically significant.

## Results

### Evolution of weight and glycemia

**Fig. 2:**
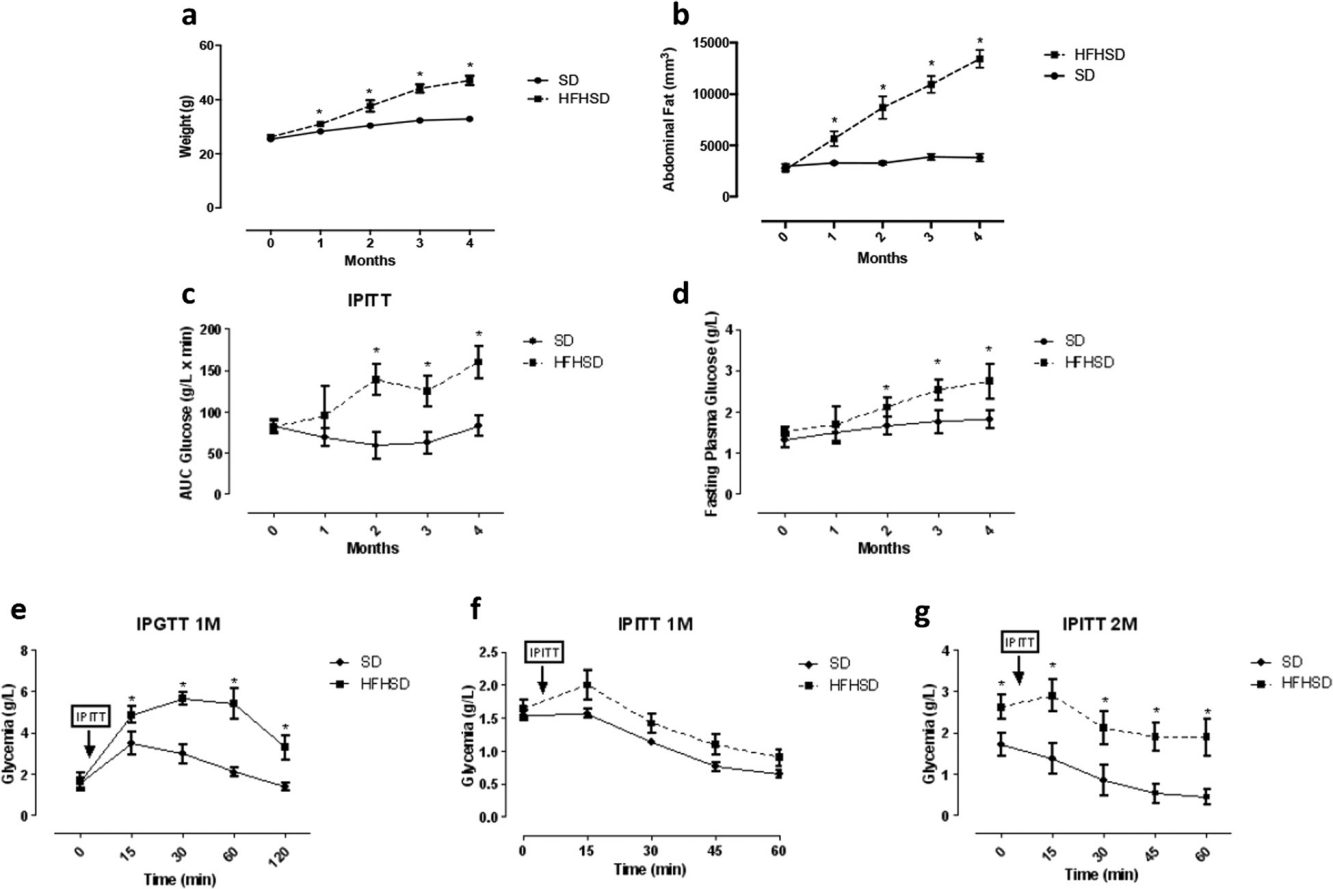
Characterization of HFHSD-induced obesity model. Mice fed the HFHSD became significantly obese (**a**) and had an increased abdominal fat volume at the first time point (**b**). Time course of intraperitoneal insulin tolerance test revealed significantly impaired glucose tolerance (**c**) and higher fasting plasma glycemia (**d**) in the HFHSD group at 2 months post-diet. Examples of intraperitoneal glucose test tolerance at 1-month (glucose administration arrow, 1 g/kg) (**e**), and intraperitoneal insulin tolerance test (insulin administration arrow, 0.75 mU/g) at 1 months (**f**) and 2-month (**g**) post diet. Data are means ± SEM. Two-way Anova test with repeated measures has been performed to assess differences *P < 0,05; **p < 0,005; ***p < 0,0005 vs SD

Mice fed with HFHSD became obese. They displayed significantly higher body weight (47.3 ± 0.7 g vs. 32.9 ± 0.1 g, *p* = 0.0006) and abdominal fat (13.4 ± 0.8 mm^3^ vs. 3.8 ± 0.4 cm^3^, *p* = 0.0002) after 4 months of diet compared to mice fed with SD. These significant weight gain and abdominal fat increase differences were already observed after one month of HFHSD (Fig. 2a and b).

HFHSD mice also displayed significantly elevated area under the curve of glucose level during intraperitoneal glucose tolerance test (IPITT) (Fig. 2c) showing impaired glucose tolerance and elevated fasting plasma glucose (Fig. 2d) at 2 months compared to SD mice. Fasting blood glucose level exceeds 2.4 g/L at 3 months (2.6 ± 1.1 g/l) in the HFHSD group suggesting type 2 diabetes status [39].

Intraperitoneal glucose tolerance test (IPGTT) showed impaired glucose secretion resulting in significantly elevated area under the curve (AUC) of glucose in the HFHSD group compared to the control group, at 1-month post diet (Fig. 2e). At the same time (1 month) AUC of glucose during IPITT was not different between HFHSD and SD mice (Fig. 2f), but became significantly higher at 2 months post-diet (Fig. 2g).

### Ectopic fat accumulation at early time points

**Fig. 3:**
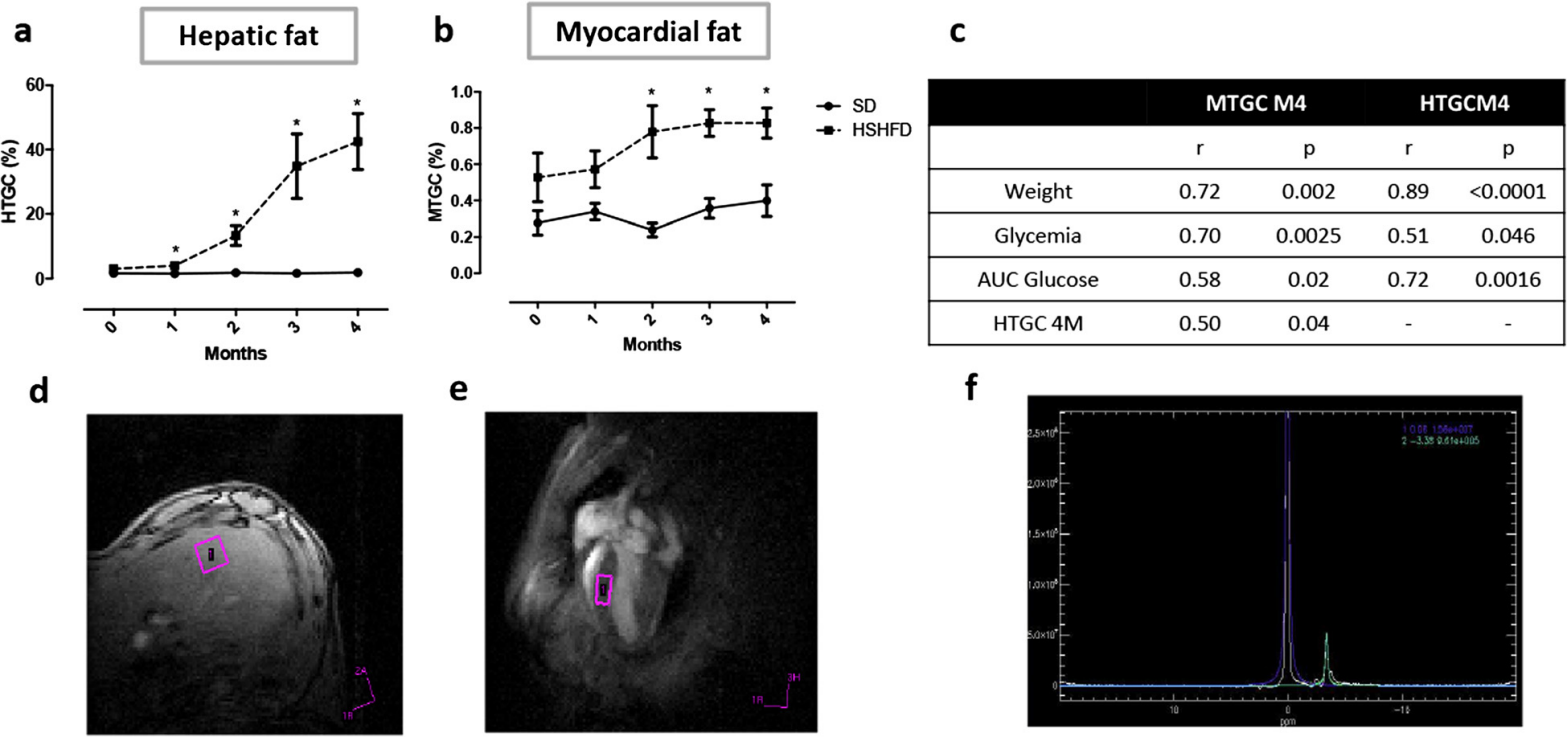
Myocardial and hepatic steatosis assessed with ^1^H-MRS. Hepatic (**a**) and myocardial (**b**) fat were followed-up during 4 months. Myocardial and hepatic steatosis were inter-correlated at M4 (**c**), and correlated to weight, glycemia and area under the curve (AUC) of glucose at M4. Volume of interest was positioned in the anterior part of the liver for hepatic triglyceride content quantification (**d**), and in the interventricular septum for myocardial triglyceride content quantification (**e**). PRESS sequence was applied to determine molecular content of water and triglyceride. Chemical shifts appear at 4.7 ppm in the spectra for water, and 1.3 ppm for triglycerides. AMARES algorithm fitting peak line shape has been used for spectra analysis (**f**). Two-way Anova test with repeated measures has been performed to assess differences. Data are mean ± SEM. *P < 0,05; **p < 0,005; ***p < 0,0005 vs SD

Triglycerides assessed with MRS were significantly higher in the liver at one month post-diet and further increased until it reached 42 ± 8 % for obese mice compared to 1.88 ± 0.27 % for SD mice at 4 months (Fig. 3a).

Although there seems to be higher myocardial triglyceride content in HFHSD groups at the beginning, this difference did not reach significant value before 2-month post-diet and remained statistically elevated until the CMR examination at 4 months in obese mice (0.82 ± 0.08 %) compared to lean mice (0.40 ± 0.09 %) (Fig. 3b). This atypical difference at baseline is probably due to variability between mice.

Myocardial and hepatic TG levels were significantly linked to weight, glycemia and AUC glucose during GTT (Fig. 3c).

Triglyceride level evaluated by biochemical assay in biopsies obtained after mice sacrifice (4-month) showed a significant higher triglyceride content in both heart and liver, which is consistent with MR spectroscopy results. (Additional file 2)

### Evolution of cardiac function during HFHSD

**Fig. 4:**
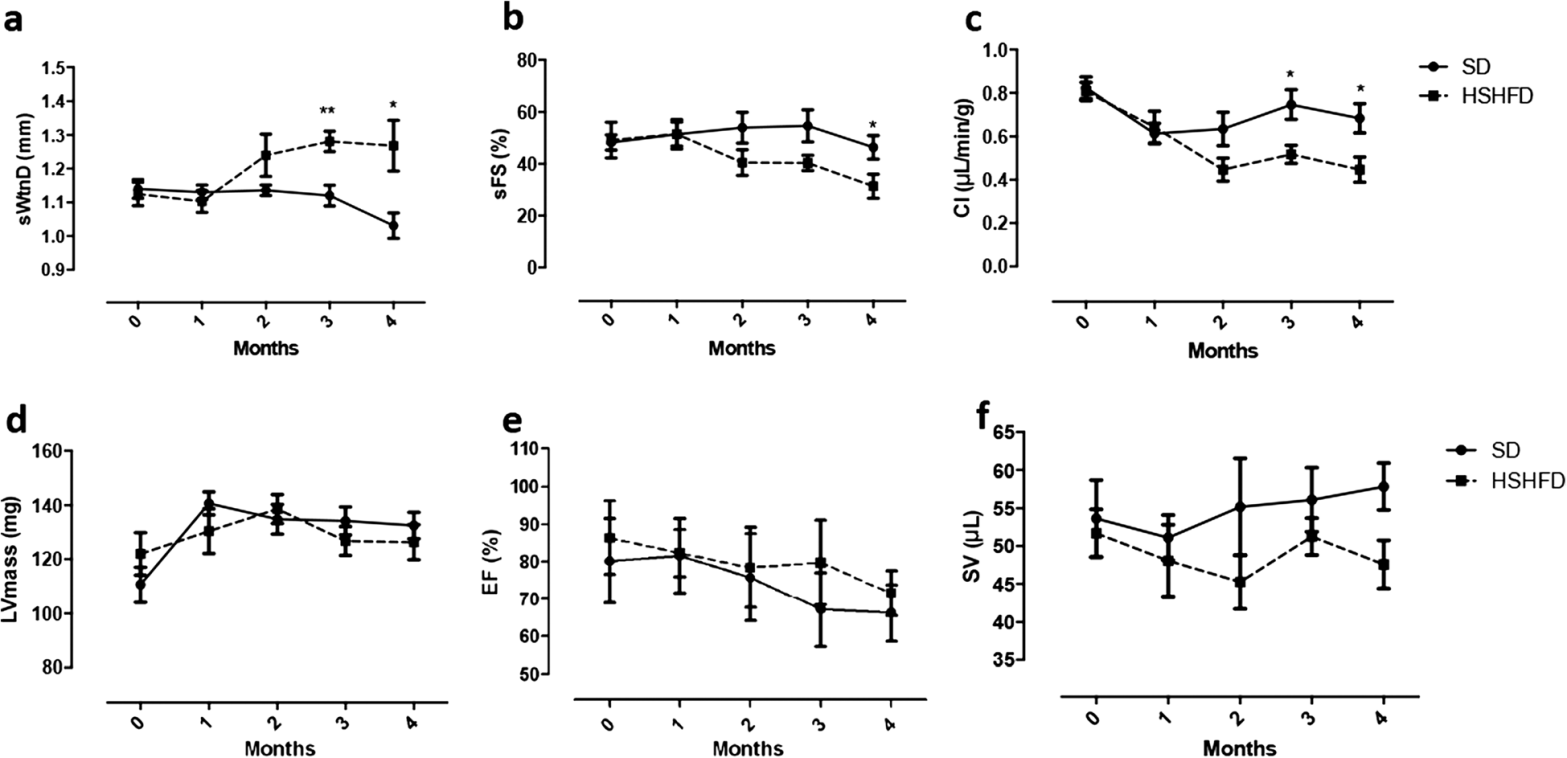
Cardiac function parameters using hemisphere cylinder model. Obese mice increased the diastolic septum wall thickness (WthD) (**a**) and decreased septum fractional shortening (sFS) (**b**) at 3-month post diet. Cardiac index (CI) was altered at the same time point (**c**). Left ventricular mass (LVmass) (**d**), ejection fraction (EF) (**e**), and stroke volume (SV) (**f**) did not change during diet. Two-way Anova test with repeated measures has been performed to assess differences. Data are mean ± SEM. *P < 0,05; **p < 0,005

HFHSD mice exhibited higher diastolic septum wall thickness (sWtnD) (1.28 ± 0.03 mm vs. 1.12 ± 0.03 mm, *p* = 0.002) (Fig. 4a) starting at 3 months post-diet compared to mice fed with a standard diet, and decreased septum percent fractional shortening (sFS) (40.3 ± 3.4 % vs. 54.6 ± 6.2 %, *p* = 0.01) at 4-month post diet (Fig. 4b).

Mice fed a HFHSD presented a significant alteration of cardiac index from 3 months post-diet compared to control mice (0.52 ± 0.04 μL/min/g vs. 0.74 ± 0.07 μL/min/g, *p* = 0.01) (Fig. 4c). This difference could be explained by significantly higher heart rate observed in HFHSD groups (data not shown). The obese group exhibited progressive reduction of cardiac index and fractional shortening during 4 months of diet compared to control mice, which, in contrast, showed constant levels of these parameters over time, together with a comparable expansion of the left ventricular mass in both groups (Fig. 4d).

Furthermore, cardiac function parameters at 4-month post-diet were correlated to both hepatic and myocardial fat content (*p* <0.05) (Table 1).

**Table 1:** Univariate analysis between ectopic lipid accumulation and cardiac parameters

	MTGC 4 M		HTGC 4 M	
	r	p	r	p
CI	- 0.60	0.02	−0.75	0.0009
sWtnD	NS	NS	0.61	0.01
sFS	NS	NS	0.61	0.01

Other cardiac function parameters such as ejection fraction and stroke volume did not change at this relatively early time point (Fig. 4e and f).

### HFHSD-fed mice displayed changes in myocardial perfusion at rest

**Fig. 5:**
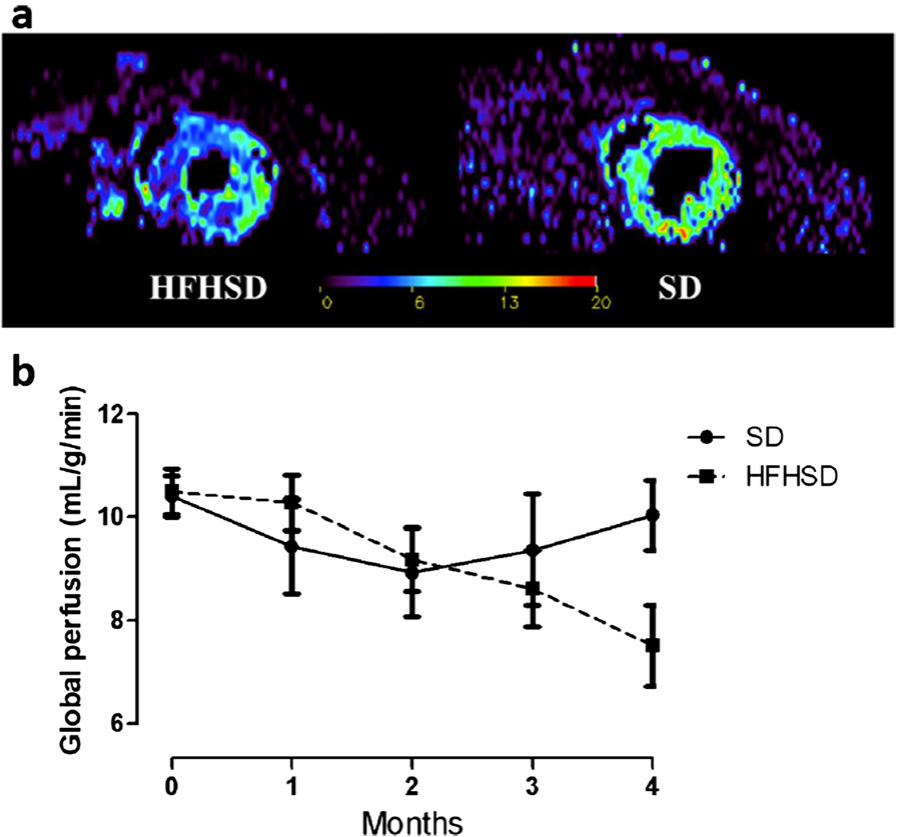
Myocardial perfusion using Cine-ASL (Arterial Spin Labeling) technique. Typical example of myocardial perfusion maps in mice fed HFHSD (left) and control mice fed SD (right) (**a**). Temporal variation in myocardial perfusion during 4 months in HFHSD and SD mouse groups (**b**). Two-way Anova test with repeated measures has been performed to assess difference *p <0,05

Anova test has not shown myocardial perfusion change during 4 months of HFHSD. However, we undoubtedly observed a significant difference in myocardial perfusion at 4-month post-diet in obese mice compared to control mice as shown in representative images (Fig. 5a). Quantitative analysis of myocardial perfusion (Fig. 5b) showed a tendency to hypoperfusion at 4-month post-diet.

Unpaired *t*-test of data at 4 months post diet alone confirmed lower perfusion observed in mice fed with HFHSD compared to mice fed with SD (7.5 ± 0.8 mL/g/min vs. 10.0 ± 0.7 mL/g/min, respectively, *p* = 0.03). Univariate analysis showed a significant correlation between myocardial perfusion and cardiac index at 4-month post-diet (r = 0.57, *p* = 0.002). Also, myocardial perfusion at 4-month was negatively associated to HTGC at the same time point (r = −0.74, *p* = 0.001). Interestingly, myocardial perfusion at 4 months post-diet was also correlated to HTGC at 3 months (r = −0.61, *p* = 0.01) and even 2 months post-diet (r = −0.64, *p* = 0.01) when no alteration in perfusion was still observed (Table 2).

**Table 2:** Univariate analysis between myocardial perfusion and ectopic fat accumulation or cardiac dysfunction

	Myocardial perfusion	
	r	P
CI 4 M	0.57	0.002
HTGC 4 M	- 0.74	0.001
HTGC 3 M	- 0.61	0.01
HTGC 2 M	- 0.64	0.01
MTGC 4 M	-	NS

**Fig. 6:**
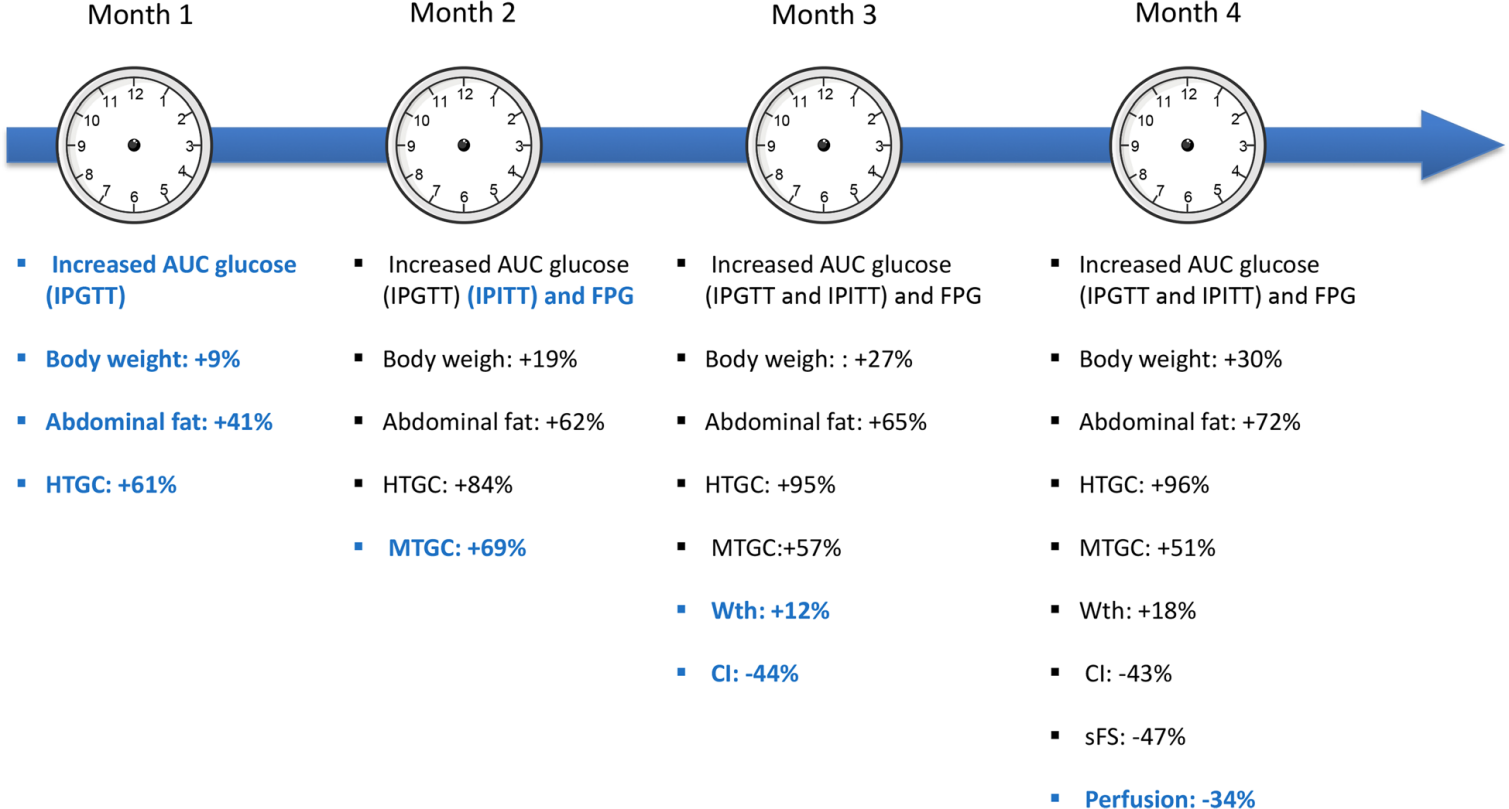
Time course of events. Time course illustration showing time points of abnormalities’ onset associated to HFHSD-induced obesity and type 2 diabetes mice model. Quantitative MR parameters allowed calculation of percentage of change compared to SD-control mice. HTGC: Hepatic triglyceride content, MTGC: myocardial triglyceride content, IPGTT: intraperitoneal glucose tolerance test, IPITT: intraperitonal insulin tolerance test, FPG: Fasting plasma glucose, Wth: Wall thickness, CI: cardiac index, sFS: Septum fractional shortening

Figure 6 has been designed to summarize time course of events observed in this study during 4 months of HFHSD.

### Exendin-4 decreased triglyceride content levels and improved cardiac function

**Fig. 7:**
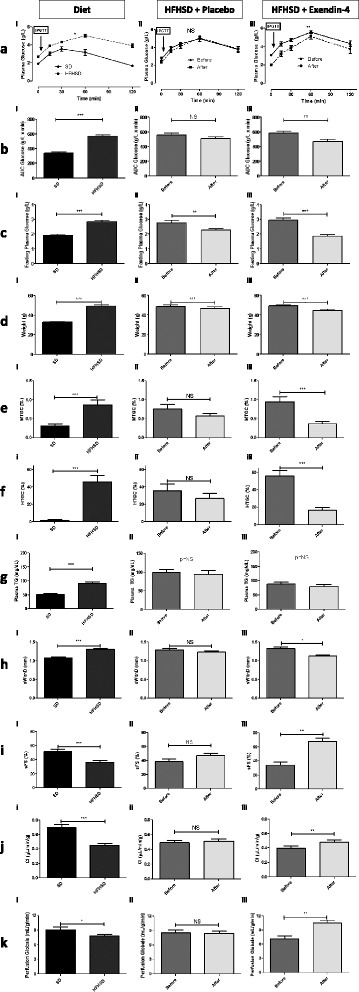
Four months diet effects on metabolic and cardiac parameters were significantly reversed by Ex-4 treatment. Intraperitoneal Glucose tolerance test showed elevated AUC glucose (**a**i, **b**i) and fasting plasma glucose (**c**i) after M4 of HFHSD. Ex-4 significantly improved glucose secretion compared to placebo mice (**a**ii, **a**iii, **b**ii, **b**iii) and reduced fasting plasma glucose in both placebo (**c**ii), and Ex-4 groups (**c**iii). Ex-4 treatment induced significant weight loss in both placebo (**d**ii) and Ex-4 groups (**d**iii) as well. Cardiac and hepatic fat accumulations are increased in obese mice (**e**i, **fi**) and short duration of Ex-4 treatment dramatically reduced both ectopic lipid accumulation (**e**iii, **f**iii) whereas no change was observed in saline-treated mice (**e**ii, **f**ii). However, Ex-4 treatment had no effect on plasma triglyceride levels (**g**ii, **g**iii) albeit significant increase 4-month post-diet in HFHSD group (**g**i). Cardiac parameters altered with HFHSD (**h**i, **i**i, **j**i) are significantly corrected after Ex-4 treatment (**h**iii, **l**iii, **j**iii) but not after saline treatment (**h**ii, **l**ii, **j**ii). sWthD: Septum wall thickness, sFS: Septum fractional shortening, CI: Cardiac index. HFHSD mice displayed hypoperfusion at 4-month (**k**i) which is completely corrected after Ex-4 treatment (**k**iii) compared to placebo group (**k**ii). Data are presented as means ± SEM. Treatment effect on AUC glucose during IPGTTT was assessed using Two-way ANOVA test. Other parameters were statistically assessed with paired *t*-test or Wilcoxon signed rank test when values were not normally distributed. *p <0,05; **p <0,005; ***p <0,0005

In the third experimental protocol, we observed significant changes of parameters at 4 months post diet, as described in the previous experimental protocol. Indeed, mice became significantly obese with increased AUC of glucose during glucose tolerance test (Fig. 7Ai, Bi, Ci and Di). They displayed higher TG accumulation both in the heart and the liver (Fig. 7Ei and Fi). These abnormalities were again accompanied by significant impairments of cardiac function parameters (Fig. 7Hi, Ii and Ji).

Furthermore, 14 days post-treatment with Ex 4 significantly decreased fasting plasma glucose and AUC glucose after GTT (Fig. 7Aii, Aiii, Bii, Biii, Cii and Ciii). Insulin level measured after sacrifice of the animals was significantly higher in HFHSD group treated with Ex-4 (2.0 ± 0.3 ng/mL) compared to placebo-treated HFHSD group (1.30 ± 0.15 ng/mL) and SD group (0.35 ± 0.05 ng/mL) (Additional file 3).

Bi-injection daily treatment with Exendin-4 (Ex-4) or Placebo resulted in a −9.6 ± 1.5 % and −4.3 ± 0.6 % significant small weight loss respectively (Fig. 7Dii and Diii). Significant higher VAT (+57 %) and SCAT (+26 %) was found in HFHSD group compared to SD group, but no change in fat mass body composition was observed after Ex-4 treatment (data not shown).

Ex-4 significantly decreased both myocardial and hepatic triglyceride content after only two weeks of treatment whereas placebo-treated mice did not (Fig. 7Eii, Eiii, Fii and Fiii). Furthermore, percentage of MTCG and HTGC decreases are significantly higher in treated mice compared to placebo (57.8 ± 4.9 % vs 2.5 ± 19.7 %, *p* = 0.02; and 71.2 ± 4.0 % vs. 0.2 ± 16.1 %, *p* < 0.0001). More interestingly, in multivariate analysis, hepatic TG decrease was independent of weight loss and glucose levels decrease and only linked to treatment (Table 3).

**Table 3:** Multiple linear regression analysis using delta HTGC and delta MTGC as the dependant variables, and delta glycemia, weight loss and treatment as independent variables

Dependant variable	Variables entered in the models	Total R	Reg. *P* value	ß coefficient	t	Sig.
Delta HTGC	Weight loss	0.76	0.0005	0.31	1.7	0. 10
	Delta AUC Glucose			0.26	1.5	0.15
	Treatment			0.41	2.0	0.05
Delta sFS	Delta AUC Glucose	0.57	0.02	−0.04	−0.2	0.85
	Treatment			−0.54	−2.6	0.018
Delta Perfusion	Delta Glycemia	0.54	0.04	−0.32	−1.4	0.18
	Treatment			−0.29	−1.3	0.23

Delta = Value before treatment – Value after treatment

Plasma triglyceride measurements in HFHSD mice showed significant higher plasma triglycerides compared to SD mice (90.87 ± 24.03 vs 52.28 ± 7.91, *p* = 0.0009) (Fig. 7Gi). Short time exendin-4 treatment had no effect on these triglycerides levels (Fig. 7Gii and Giii)

Exendin-4 treatment significantly reduced the septal thickness (Fig. 7Hii and Hiii) and increased the fractional shortening (Fig. 7Iii and Iiii). The decrease in septal thickness was correlated to treatment effect only (r = 0.42, *p* = 0.04), and delta fractional shortening was independently associated to this treatment (Table 3).

We observed a strong alteration of the cardiac index at 4-month post-diet (0.48 ± 0.04 μL/min/g in obese mice vs. 0.72 ± 0.05 μL/min/g in control mice; *p* < 0.0001) (Fig. 7Ji). Impaired cardiac index observed in HFHSD group was partly reversed by Ex-4 treatment (Fig. 7Jii and Jiii) but remained significantly higher than in the control group (*p* = 0.0005). Linear regression shows no link between weight loss and CI improvement, or decrease in AUC of Glucose.

We also found significant changes in myocardial perfusion at rest 4-month post diet, as observed in the first part of the study at the 4-month time point analyzed using unpaired *t*-test (Fig. 7Ki). Finally, short duration of Ex-4 treatment also increased myocardial perfusion and reached the SD group level (Fig. 7Kii and Kiii). Delta perfusion was significantly correlated to treatment effect and to glycemia improvement, but not to weight loss (Table 3).

## Discussion

Multimodal CMR allows the simultaneous evaluation of different parameters and the correlation with different information on specific cardiac pathologies. In human studies it is possible to assess these parameters at one time point and to follow changes in time, but it is difficult to study the development of the pathology from early to late stage. Animal models offer the ability to do such follow-ups in a well-monitored evolution of the disease. Here, we performed a robust multi-modal in vivo MR protocol in mice including simultaneous assessment of cardiac function, myocardial perfusion and quantification of lipid accumulation in both heart and liver within reasonable scan- and post-processing-time. We showed early-elevated hepatic triglyceride content at one-month post-HFHSD, followed by elevated myocardial triglyceride content at two months. Impairments of cardiac function and myocardial perfusion were measurable at 3 and 4 months. Finally, these abnormalities were partially or totally reversed after 14-day treatment with Exendin-4.

Both intra-cardiac and intra-hepatic triglyceride content have been validated against biochemical assay by others [17, 40]. In this current study, we verified that the difference in TG content in the heart and the liver between HFHSD and SD groups shown by MR spectroscopy agreed with difference observed using in vitro biochemical assay measurement. Furthermore, our ^1^H-MRS and biochemical estimates are consistent with values reported in other studies [41–43].

To our knowledge, there is no in vivo animal study investigating both heart and liver lipid content simultaneously in the context of the development of obesity and diabetes.

Weight follow-up, glucose tolerance test and insulin tolerance test confirmed that the HFHSD mice developed obesity and impairment of glucose homeostasis within the experimental time course when compared to SD mice as described by others [44]. Both cardiac and liver steatosis in obesity and diabetes have been described in human and animal studies [9–11], highlighting the potential effects of these conditions on the heart. However little is known on the time course of TG accumulation in heart and liver during the development of the disease and on their correlation with myocardial perfusion and function. Our longitudinal MRS analysis showed early TG accumulation in liver and heart. Moreover, TG accumulation in the liver preceded that of the heart suggesting pivotal involvement of hepatic insulin resistance in the development of metabolic complications of obesity. Another relevant observation is that although we found a significant correlation between both hepatic and cardiac fat accumulation, linear regression showed an important disparity between both tissue TG accumulations. These combined data strengthen our hypothesis regarding a tissue specific mobilization of ectopic fat development based on data obtained in patients. Indeed, obese patients have different phenotype of ectopic fat accumulation. While some patients exhibit elevated hepatic triglyceride content and normal pancreatic triglyceride content level, others have increased cardiac triglyceride content with normal hepatic triglyceride content. This indicates tissue-specific distribution of TG in non-adipose tissue.

Furthermore, in our previous studies, we assessed ectopic fat modulation after weight loss in obese patients who underwent bariatric surgery. Ectopic fat accumulation did not respond equally. We observed a huge decrease of hepatic and pancreatic triglyceride contents [10], accompanied by a moderate epicardial adipose tissue reduction, and no change in myocardial triglyceride content [45]. Moreover, percentages of ectopic fat losses were neither correlated with each other, nor with body mass change. This observation has also been described by others [46], [47], which consolidates our hypothesis.

As expected, myocardial and hepatic TG levels are significantly linked to weight, glycemia and AUC glucose during IPGTT. Negative correlation of both hepatic and myocardial triglyceride content with cardiac function was observed in our study. This result is in agreement with other observations [48–50], and suggests that a potentially lipotoxic effect of steatosis may contribute to subclinical alteration of myocardial function. However, we also found a significant association between perfusion and TG accumulation unlike Korosoglou's findings [49].

The exact mechanisms by which steatosis causes cardiac dysfunction is unclear, however an increasing number of reports suggest that lipotoxicity results from imbalance between cardiac lipids uptake and β-oxidation. Persistent increase of β-oxidation due to excess lipids availability may lead to toxic intermediate lipid intermediates production such as ceramides and diacylglycerol, which could interfere with cardiac signaling cascade and induce cell apoptosis [51–53]. These early abnormalities induced greater reactive oxygen species production [54, 55]. In this oxidative stress environment combined with increased inflammation [56, 57], calcium homeostasis could be affected resulting in excitation-contraction coupling alteration and therefore to myocardial contractile dysfunction [58]. Increased intermyocellular fibrosis resulting from collagen stacked in extra-cellular matrix is frequently associated to cardiac steatosis observation, and could be responsible for tissue stiffness, reducing diastolic relaxation [59, 60]. In the longer term, cardiac energetics could be affected as well, accompanied by inefficient mitochondrial ATP production [61]. Involvement of the renin-angiotensin system and more recently micro-RNAs has also been described [56, 62–64].

Cine MRI showed late alteration of systolic function as reported by Nagarajan et al. [65], and others [6, 13, 66, 67]. However, conflicting results exist in the literature regarding early cardiac dysfunction in metabolic alterations [68–70]. Differences in experimental conditions such as the animal model, the duration of experiments, or the diet composition may explain these discrepancies. It would be interesting to delve into cardiac function in this model with the measurement of other parameters such as mitral peak velocity [68, 69] and regional myocardial function as well (strain, torsion, and synchrony) which may be reduced earlier, before impairment of global cardiac parameters [71]. We did not explore regional cardiac dysfunction, but we measured the thickness of different parts of the myocardial wall as described by others [72] and found a significant higher wall thickness in mice fed a HFHSD.

Myocardial perfusion was quantified using a strictly non-invasive arterial spin labeling technique that could be applied in each mouse repetitively in a reduced scan time [28, 73]. Although no significant difference was observed using two way ANOVAs test with repeated measures which includes time effect in the follow-up study statistical analysis; a significantly diminished myocardial perfusion in HFHSD mice was observed at 4-month in experimental protocol 3. This difference is consistent with the result obtained when the 4-month time point is analyzed alone in experimental protocol 2. Previous studies on small animals have examined myocardial perfusion at baseline and under various conditions including myocardial infarction [74], vasodilator stress [75], anesthesia [20] transgenic mice [76] and in non-obese diabetic rat hearts [77]. In a recent study, Naresh NK et al. have used first-pass MBF measurements under rest and regadenoson stress in a high-fat diet obesity mouse model [78]. They found no difference in myocardial blood flow (MBF) at rest between control and obese mice, but evidenced a reduced adaptation of flow during stress. In our study, we performed a longitudinal assessment of myocardial perfusion during 4 months of HFHSD and showed a significant alteration of basal perfusion, but under isoflurane anesthesia. Isoflurane anesthesia is a known vasodilator and might have caused herein a light stress condition eliciting perfusion differences between controls and HFHSD animals [79]. This is supported by the fact that all perfusion values found in this study are comparatively high. The reduced perfusion measured under isoflurane may therefore be due to endothelial dysfunction in response to interrelated chronical ROS production, inflammatory state, reduced nitric oxide bioavailability, and ceramides [80–83]. Microcirculation remodeling implying possible interstitial or perivascular fibrosis as shown in humans by Chiribiri [84] could be also involved. Additional investigations including pharmacologic stress will be useful for a complementary understanding of microvascular functional defects in diabetic cardiomyopathy.

In the second protocol, we studied the short-term effect of Exendin 4 treatment on ectopic fat depots development and cardiovascular alterations. Exendin-4, a glucagon-like peptide-1 (GLP-1) receptor agonist is among those treatments of type 2 diabetes that lower blood glucose level by increasing glucose-dependent insulin secretion and suppressing excess glucagon secretion [85]. It reduces weight as well by inducing satiety. GLP-1 and GLP-1 receptor (GLP-1R) agonists are considered as beneficial for cardiovascular function [28–30, 86, 87] given that they improve biomarkers of cardiovascular risk, decrease systolic blood pressure, improve endothelial function and display beneficial actions on acute ischemic myocardial damage [88].

Placebo and ex-4 treated mice groups displayed a significant weight loss after 15 days of treatment with no change in diet composition. This effect seems to be mainly due to stress-induced by bi-injection daily rather than to treatment, especially in placebo group.

Our work demonstrates that a short treatment with Exendin-4 improves myocardial function parameters and increases myocardial perfusion. This effect was linked to treatment and decreased glycaemia throughout multivariate analysis.

Fractional shortening and cardiac perfusion increased beyond the level of SD controls. Significant overcompensation has been verified by statistical analysis of GLP-1 response versus SD levels. Comparison between HFHSD post-GLP-1 treatment with SD groups showed no significant difference in myocardial perfusion (*p* = 0.12). By contrast, fractional shortening was significantly higher after GLP-1 treatment compared to SD controls (*p* = 0.009). We don’t have any specific explanation for this observation.

A comparable beneficial effect of GLP-1 on vascular function was previously shown in peripheral vessels, when GLP1 administration enhanced insulin mediated forearm blood flow responses to ACh [28, 89]. In swine, GLP-1 infusion during 4 h attenuates post-resuscitation myocardial microcirculatory dysfunction [90]. In humans, the effect of GLP-1 agonist on perfusion is debated. In healthy overweight volunteers, acute exenatide infusion increases capillary perfusion via nitric oxid independent pathway [91]. In a small study of eight T2D male without coronary artery disease, exenatide induced an increase in myocardial blood flow evaluated by PET, without changing myocardial glucose uptake [92]. Nevertheless, this effect is missing in Faber R’s work [93].

We found also that short-term GLP-1 treatment drastically diminished both cardiac and hepatic steatosis whereas it had no effect on plasmatic triglycerides. This important effect after such a short administration is impressive and contrasts with the small impact of the treatment on both weight and glycemia. This result emphasizes the data of Monji et al. who reported that 40 days treatment with Exendin-4 reduced ceramide staining in two mouse models of diabetes [32].

The impact of GLP-1 treatment in cardiac steatosis has not been evaluated in humans. Furthermore, there is limited information on the impact of GLP-1 analog on real life patient [94], and the long term effect of GLP-1 agonist in diabetic cardiomyopathy is unknown.

## Conclusion

To our knowledge, this is the first longitudinal study involving CMR multimodal imaging in diet induced obese mice showing early increase in hepatic and cardiac steatosis preceding alteration of perfusion and cardiac function. Importantly, we have demonstrated a beneficial effect of Exendin-4 on cardiac parameters and both hepatic and cardiac steatosis. The sequence of events elicited in the development of the cardiometabolic disease in mice as well as the effect of exendin-4 on cardiac steatosis are of potential interest to build diagnostic and therapeutic strategies in humans.

## Additional files

Additional file 1:
**High Fat High Sucrose Diet (U8978 version 19 SAFE).** (PDF 331 kb) Additional file 2:
**Biochemical measurements of triglyceride in myocardial and hepatic tissue.** TG assessment in heart and liver tissue using biochemical assay showed significantly higher TG content in mice fed a HFHSD compared to mice fed a SD. Unpaired *t* test has been performed to assess differences. **P* < 0,05; ***p* < 0,005. (PDF 153 kb) Additional file 3:
**Plasma insulin level quantification: Insulin measurement using Elisa kit showed higher insulin level in HFHSD mice group treated with Ex-4 compared to HFHSD mice group injected with placebo, and SD mice group.** One-way Anova test has been performed to assess differences. **P* < 0,05; ****p* < 0,0005. (PDF 164 kb)

## Abbreviations

ASL: Arterial spin labeling
AUC: Area under the curve
CI: Cardiac index
ECG: Electrocardiogram
EDV: End diastolic volume
ESV: End systolic volume
GLP-1: Glucagon-like peptide-1
H-MRS: Proton magnetic resonance spectroscopy
HFHSD: High fat high sucrose diet
HOMA-IR: Homeostatic model assessment of insulin resistance
IPGTT: Intra-peritoneal glucose test tolerance
LV: Left ventricle
MBF: Myocardial blood flow
MR: Magnetic resonance
CMR: Cardiac magnetic resonance
MRI: Magnetic resonance imaging
PRESS: Point resolved spectroscopy sequence
SC BID: Subcutaneously Bi injection daily
SD: Standard diet
sFS: Septum fractional shortening
SV: Stroke volume
sWtnD: Septum wall thinckness at diastole
T: Tesla
TG: Triglycerides
TR: Repetition time
TE: Echo time
NA: Number of averages
