# Supplementary material for: Time course of cardiometabolic alterations in a high fat high sucrose diet mice model and improvement after GLP-1 analog treatment using multimodal cardiovascular magnetic resonance
Source: J Cardiovasc Magn Reson. 2015 Nov 6;17:95. doi: 10.1186/s12968-015-0198-x (PMC4636800; doi:10.1186/s12968-015-0198-x)
Supplement: Additional file 1: — High Fat High Sucrose Diet (U8978 version 19 SAFE). (PDF 331 kb) [file 12968_2015_198_MOESM1_ESM.pdf]

High Fat High Sucrose Diet (U8978 version 19 SAFE)

Casein	22.8%
DL Methionine	0.2%
Maltodextrin	17.15%
Sucrose	16.633%
Anhydrous butter	33.35%
Soja Oil	2.5%
AIN mineral 93G-mx	4.55%
Sodium bicarbonate	1.05%
Potassium citrate	0.4%
AIN Vitamin 93-vx	1.3%
Choline bitartrate	0.2%
Anti oxydant	0.0028%
