# Supplementary figures and images for: Time course of cardiometabolic alterations in a high fat high sucrose diet mice model and improvement after GLP-1 analog treatment using multimodal cardiovascular magnetic resonance
Source: J Cardiovasc Magn Reson. 2015 Nov 6;17:95. doi: 10.1186/s12968-015-0198-x (PMC4636800; doi:10.1186/s12968-015-0198-x)

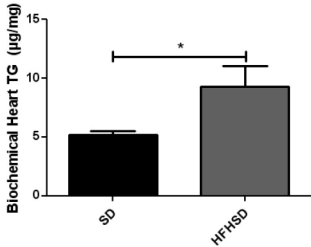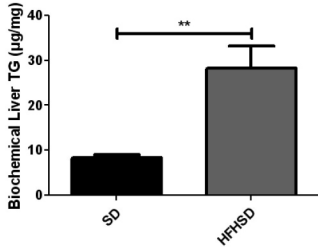

Supplement: Additional file 2: — Biochemical measurements of triglyceride in myocardial and hepatic tissue. TG assessment in heart and liver tissue using biochemical assay showed significantly higher TG content in mice fed a HFHSD compared to mice fed a SD. Unpaired t test has been performed to assess differences. *P < 0,05; **p < 0,005. (PDF 153 kb) [file 12968_2015_198_MOESM2_ESM.pdf]

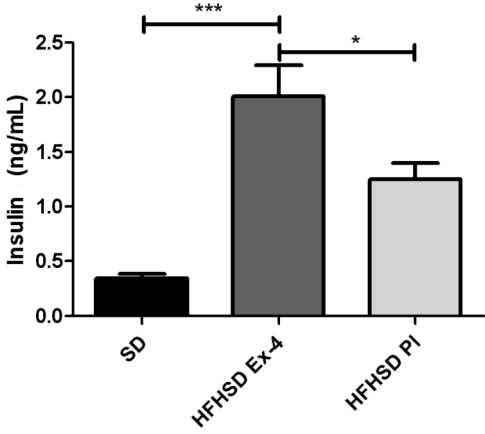

Supplement: Additional file 3: — Plasma insulin level quantification: Insulin measurement using Elisa kit showed higher insulin level in HFHSD mice group treated with Ex-4 compared to HFHSD mice group injected with placebo, and SD mice group. One-way Anova test has been performed to assess differences. *P < 0,05; ***p < 0,0005. (PDF 164 kb) [file 12968_2015_198_MOESM3_ESM.pdf]
